# Physiological and intestinal microbiota responses of sea cucumber *Apostichopus japonicus* to various stress and signatures of intestinal microbiota dysbiosis

**DOI:** 10.3389/fmicb.2024.1528275

**Published:** 2024-12-23

**Authors:** Liang Cui, Yumeng Xie, Kai Luo, Mingyang Wang, Longzhen Liu, Changlin Li, Xiangli Tian

**Affiliations:** ^1^The Key Laboratory of Mariculture, Ministry of Education, Ocean University of China, Qingdao, China; ^2^The Yellow Sea Fisheries Research Institute, Chinese Academy of Fishery Sciences, Qingdao, China

**Keywords:** environmental stress, immune responses, intestinal microbiota, dysbiosis, stress assessment, *Apostichopus japonicus*

## Abstract

Identifying the signatures of intestinal dysbiosis caused by common stresses is fundamental to establishing efficient health monitoring strategies for sea cucumber. This study investigated the impact of six common stress experienced frequently in aquaculture on the growth performance, intestinal homeostasis and microbiota of sea cucumber, including thermal (23°C), hypoosmotic (22‰ salinity), ammonium (0.5 mg/L NH_4_^+^-N), and nitrite (0.25 mg/L NO_2_^−^-N) stress exposure for 30 days, as well as starvation and crowding (6 kg/m^3^ density) stress exposure for 60 days. Results demonstrated that all stress led to reduced growth performance and digestive capacity of sea cucumber, along with varying degrees of oxidative stress and immune responses. Various stresses significantly altered the diversity, community structure (except for crowding stress), and composition of intestinal microbiota. The ratios of Bacteroidota: Proteobacteria (B: P) and Firmicutes: Proteobacteria (F: P) declined markedly compared to the control. Potentially pathogenic bacteria of Shewanellaceae, Vibrionaceae, and Moraxellaceae significantly increased under crowding, ammonium, and nitrite stress, respectively, whereas beneficial microbes of *Achromobacter* and Rhodobacteraceae were, respectively, enriched under hypoosmotic and starvation stresses. The complexity and stability of microbial ecological networks were further altered by these stresses. KEGG predictions revealed the reduced functional pathways of intestinal microbiota involved in host immunity under different stresses. Correlation analysis further confirmed a strong link between microbiota response and host immunity under different stresses. The increased abundance of Verrucomicrobia species could also be identified as the sensitive indicator for diagnosing whether the host was under stressful pressure by random forest analysis.

## Graphical abstract

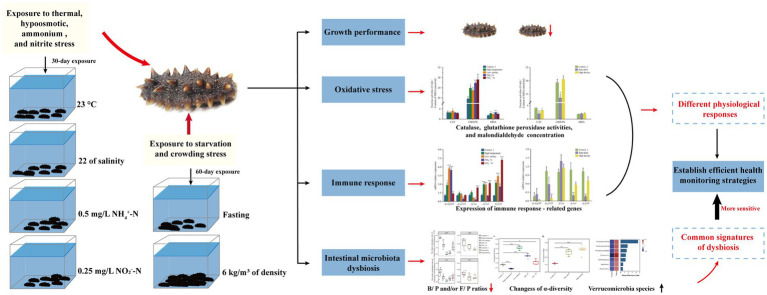

## 1 Introduction

As the global population continues to rise and the demand for sustainable aquaculture increases, the industry is challenged to enhance production while conserving energy and reducing emissions, hence the intensive culture methods are rapidly developing (El-Saadony et al., 2022). Although this farming model has temporarily boosted aquaculture productivity and economic benefits, it has also led to environmental pressures such as water quality deterioration and spatial limitations that weaken the immune function of aquatic animals, promote disease spread, and lead to frequent outbreaks of diseases in aquatic animals (Assefa and Abunna, 2018; Legrand et al., 2020). However, the disease outbreaks under environmental pressures are often closely associated with alterations and imbalances in the host’s intestinal microbial community (Xiong et al., 2015). The intestinal microbiota in aquatic animals, existing in a state of dynamic equilibrium, is crucial for maintaining host health, particularly through the facilitation of immune system development, immune response regulation, and pathogen colonization inhibition, while, disturbances from environmental stress in aquaculture can induce microbiota dysbiosis, leading to pathogen invasion and subsequent disease outbreaks (Huang et al., 2018; Rowland et al., 2018). Especially, this disturbance in the intestinal microbiota (microbiota dysbiosis) will further increase the host’s susceptibility to diseases (Petersen and Round, 2014). Therefore, due to the close relationship between the instability of the intestinal microbial community and host health, efforts have been made to monitor the health of aquatic animals by identifying the signatures of intestinal microbiota dysbiosis in response to diverse environmental stresses, with the aim of timely *Bacteroidetes* microbiota homeostasis to reduce host morbidity (Xiong et al., 2019).

Currently, the signatures of intestinal microbiota dysbiosis in aquatic animals under various aquaculture environmental pressures have been extensively explored. Specifically, alterations in water quality parameters in aquaculture (e.g., temperature, salinity, ammonium, and nitrite) have been shown to impact the diversity and composition of intestinal microbial community in aquatic organisms (Huang et al., 2018; Wang et al., 2021). A previous study has reported that the species evenness (Shannon index) of the hind-intestine microbial community in sea cucumbers (*Apostichopus japonicus*) significantly decreases under high temperature (20°C), high concentrations (50 mg/L) of ammonium or nitrite stresses (Zhang Z. et al., 2019). Under elevated salinity conditions (30 practical salinity units), there was an observed increase in the abundance of potential pathogens such as *Photobacterium* and *Pseudoalteromonas*, coupled with a reduction in the abundance of beneficial bacteria including the *Bacteroidetes* in the intestines of *Litopenaeus vannamei* (Zhang et al., 2016). Furthermore, the environmental stresses (e.g., inadequate culturing space and food) resulting from inappropriate culturing practices has been shown to impact the composition and function of the intestinal microbial community (Dai et al., 2018; Zheng et al., 2023). For instance, the *Mycoplasma*, a potential pathogen, was notably enriched in the intestines of juvenile *Micropterus salmoides* under high culturing density stress (160 fish/m^3^) (Zheng et al., 2023). Under starvation stress, a shift from Firmicutes to Proteobacteria was observed in the dominant phylum in the intestines of leopard coralgrouper (*Plectropomus leopardus*), and the functional pathways related to nutrient metabolism of the intestinal microbial community in starved *L. vannamei* were enhanced (Dai et al., 2018; Mekuchi et al., 2018). These findings illustrate the distinct response patterns of intestinal microbiota in aquatic animals under various environmental stresses, which is crucial for enabling early detection and intervention prior to the host progressing to an irreversible disease state by identifying the prevalent indicators of intestinal microbiota dysbiosis (Xavier et al., 2023).

Sea cucumber has emerged as an important pivotal species in mariculture in Asian countries like China, Japan, and South Korea, owing to its considerable nutritional and economic value (Han et al., 2016). As a stenohaline temperate marine species, sea cucumbers have stringent requirements for water quality conditions, including temperature and salinity, and are highly susceptible to disturbances from toxic factors (e.g., ammonium and nitrite) and other aquaculture environmental stresses (e.g., crowding and starvation stress) (Han et al., 2016; Tian et al., 2014; Zhang et al., 2018; Zhang Z. et al., 2019). Accordingly, it is imperative to identify the signatures of intestinal microbiota dysbiosis in sea cucumbers under various aquaculture stresses to dynamically monitor the host’s health status and prevent disease outbreaks. However, the majority of current studies predominantly focus on examining the characteristics of intestinal microbiota dysbiosis in sea cucumbers under different levels of single environmental stress (Pei et al., 2024; Zhao et al., 2019; Zhao Y. et al., 2023; Zhao Z. L. et al., 2023), while lacking investigations into common features of intestinal microbiota dysbiosis under diverse perturbations (Zhang Z. et al., 2019).

Thus, the growth performance, oxidative stress levels, and immune defenses of sea cucumber under various common stresses in aquaculture environments (thermal, hypoosmotic, ammonium, nitrite, crowding, and starvation stress) will be detected to identify the changes in growth and physiological responses of sea cucumber under each aquaculture stress in this study. Moreover, the signatures of intestinal microbiota dysbiosis, along with the crosstalk effect between alterations in the intestinal microbiota and nonspecific defenses in sea cucumbers under different aquaculture pressures will be further explored based on the 16S rRNA gene amplicon sequencing analysis. The findings of this study will advance the comprehension of interplay between intestinal microbiota homeostasis and the health of aquatic organisms, providing reliable indicators for the dynamic surveillance of intestinal microbiota homeostasis in sea cucumber.

## 2 Materials and methods

### 2.1 Animal culture and stress exposure

A two-week acclimation period was employed in the 1 m^3^ water tanks to familiarize the sea cucumbers with the laboratory culturing environment. During this period, the water with continuous aeration and temperature was kept at 17 ± 1°C, salinity at 30 ± 1‰, pH at 7.8–8.1, and ammonium and nitrite concentrations below 0.05 mg/L, with a stocking density of 2 kg/m^3^. Subsequently, the sea cucumbers underwent a 24 h fasting period, after which 400 healthy sea cucumbers (average weight of 18.07 ± 0.13 g) were randomly placed into eight groups and each treatment had five replications, each aquarium (60 × 30 × 40 cm) curing 8 sea cucumbers except for the High density group (comprising 24 sea cucumbers). The six treatment groups consisted of a 30-day pressuring period High temperature group (culturing temperature at 23°C), Low salinity group (culturing salinity at 22), NH_4_^+^-N group (co-cultured with 0.5 mg/L NH_4_^+^-N, by adding NH_4_Cl), NO_2_^−^-N group (co-cultured with 0.25 mg/L NO_2_^−^-N, by adding NaNO_2_), as well as a 60-day pressuring period Starvation group (fasted for 60 days) and High density group (culturing density of 6 kg/m^3^). Additionally, there were control groups for culturing 30 days (Control_1 group) and 60 days (Control_2 group). The specific stressful conditions for each group were determined based on relevant research (Dong et al., 2005; Hu et al., 2012; Luo et al., 2014; Wang et al., 2014; Wang et al., 2017). During the experiment, all rearing conditions remained consistent with the acclimation period, except for the varying stressful conditions in different treatment groups. Daily, the culturing water was replaced at a set time (16:00), residual feed and feces were gathered before each water exchange, and the sea cucumbers were provided with feed (a mixture of 40% commercial feed and 60% marine sediment) after the water change.

### 2.2 Sampling and growth performance

After the stress exposure finished, sea cucumbers were weighed and sampled following a 24 h fasting period. Three sea cucumbers were randomly selected from each aquarium and dissected under sterile conditions. The coelomic fluid, mid-intestine, and hind-intestine samples collected from three sea cucumbers in each aquarium under sterile conditions were, respectively, combined into individual parallel samples, and promptly stored at −80°C (no more than 1 month) for subsequent analysis. Because *A. japonicus* is not an endangered or protected species, no permission was needed for animal collection, and an ethics statement is not applicable.

Furthermore, the corresponding growth performance and survival rate of sea cucumber were calculated by referring to the following formulas:


$$\text{Survival rate}\;\left(SR\right)=S_{2}/S_{1}\times 100\%$$

$$\text{Specific growth rate}\;\left(SGR\right)=100\%\times \left[\left(\ln\;W_{2}-\ln\;W_{1}\right)/T\right]$$

$$\text{Weight gain rate}\;\left(WGR\right)=\left(W_{2}-W_{1}\right)/W_{1}\times 100\%$$

$$\text{Feed efficiency}\;\left(FER\right)=100\%\times \left(W_{2}-W_{1}\right)/C$$

$$\text{Apparent digestibility rate}\;\left(ADR\right)=100\%\times \left(C–F\right)/C$$

In these formulas, S1 and S2 represent the initial and final number of the sea cucumbers, W1 and W2 represent the initial and final weights of the sea cucumbers, T represents the duration of the experiment (30/ 60 days), C represents the dry weight of the ingested feed, and F represents the dry weight of the feces.

### 2.3 Enzyme activity assays

The coelomic fluid was centrifuged at 5000 rpm at 4°C for 15 min to collect the supernatant (CS). The CS was then analyzed for oxidative stress levels including the activities of catalase (CAT) and glutathione peroxidase (GSH-PX), and malondialdehyde (MDA) concentration by the assay kit methods (Nanjing Jiancheng Bioengineering Institute, China). The specific assay procedures strictly adhered to the instructions provided with the assay kits (A007-1-1, A005-1-1, A003-1-1).

### 2.4 Quantification of gene expression

Total RNA was isolated from mid-intestine tissues utilizing the Trizol method (Ambion, United States). After evaluating the integrity, purity, and concentration through agarose gel electrophoresis and a NanoDrop2000 micro-spectrophotometer (NanoDrop Technologies, Wilmington, United States), the high-quality RNA was reverse transcribed into cDNA, and the expression levels of immune-related genes (including *Aj-p50*, *Aj-p105*, *Aj-rel*, *Aj-lsz*, *Aj-hsp90*) and the reference gene (*β-actin*) in the intestinal tissues were further determined through reactions of real-time quantitative PCR, and specific primer sequences for those genes referred to from previous studies are provided in Supplementary Table S1 (Liu et al., 2022; Xu et al., 2024). Primer specificity was verified through a melting curve analysis, and the relative expression levels of each target gene were calculated based on the analysis of the cycle threshold values (Livak and Schmittgen, 2001).

### 2.5 Intestinal microbiota analysis by Illumina MiSeq sequencing

Genomic DNA of the intestinal microbiota from the hind intestine was obtained using PowerFecal DNA Extraction Kit (Mobio, Carlsbad, United States). Subsequently, the V3–V4 fragment of 16S rRNA gene was specifically amplified using the universal primers 343F (5’-TACGGRAGGCAGCAG-3′) and 798R (5’-AGGGTATCTAATCCT-3′), and the amplification system and reaction protocol were referred from the previous study (Xie et al., 2023). The evaluated and purified PCR products were further submitted to the Illumina MiSeq sequencing platform for sequencing.

Raw sequencing data was processed and implemented in QIIME software, including quality filtering, denoising, merging, and chimera removal. After quality control, effective sequences were obtained and grouped into operational taxonomic units (OTUs) with a 97% similarity threshold. Moreover, representative sequences within the OTUs were annotated based on the SILVA 138 database. To mitigate diversity biases arising from sequencing depth, the sequencing depth of all samples was standardized.

### 2.6 Bioinformatic data analysis

Invsimpson index was computed through QIIME (version 1.9.1) software (Bolyen et al., 2019). Subsequently, R software (version 4.3.1) was employed for comprehensive sequencing data analysis. The “Vegan” R package was employed to conduct the analysis of beta diversity, utilizing the “adonis” and “anosim” functions to carry out permutational multivariate analysis of variance (PERMANOVA) and similarity analysis based on Bray-Curtis distance, with 1,000 permutational test for each test. Principal coordinate analysis (PCoA) was utilized to visualize the differentiation among microbial samples (Dixon, 2003). Indicator species in each group were identified using “indicspecies” R package. Differential taxa between stress groups and control groups were investigated using Linear discriminant analysis Effect Size (LEfSe). Spearman and Pearson correlation analyses were primarily conducted using the “psych” R package, and the correlation heatmap was further visualized using the “pheatmap” R package. Functional predictions of the gut microbiota were performed using PICRUSt2 software, and the relative abundances of the top 34 metabolic pathways at KEGG level 2 were visualized using the “pheatmap” R package (Douglas et al., 2020). The random forest model was constructed using the “RandomForest” and “dplyr” R packages, the variable importance was further evaluated by “rfPermute” R packages, the model evaluation parameters were calculated and adjusted using the “caret” R package, the classification model and feature importance ranking results were further exported, and the performance of the classification model was assessed using a confusion matrix (Edwards et al., 2018). Network analysis was performed on OTUs with relative abundances greater than 0.1%, selecting those strong correlationships (Spearman correlation analysis, |r| ≥ 0.8 and *p* < 0.05) to construct a molecular ecological network. The “igraph” R package was utilized to output network node and edge features, and the Gephi software (version 0.10.1) was employed for network visualization. Network topology attributes, modularity features, and stability features were also computed and visualized using “igraph” and “ggClusterNet” R packages (Li et al., 2019; Wen et al., 2022; Zhu et al., 2019).

### 2.7 Statistical analysis

The experimental data were processed by IBM SPSS 24 software, and all data were presented as mean values ± standard error (S.E.). Student’s *t*-test was performed to compare the differences between the control group and various environmental stress groups, with a significance level set at *p* < 0.05 to determine the significance of inter-group differences.

## 3 Results

### 3.1 Growth performance

In this study, exposure to different environmental stressful conditions significantly decreased the growth (WGR and SGR) and digestive performance (ADR and FER) of sea cucumber (*p* < 0.05), with no observed mortality across the diverse stress groups (Table 1). Specifically, during the 30-day culture period, sea cucumbers exposed to thermal, hypoosmotic, excessive ammonium, and nitrite stress exhibited significantly lower WGR, SGR, ADR, and FER than the Control_1 group (*p* < 0.05). Similar trends were also evident in the 60-day cultured groups: sea cucumbers subjected to starvation stress exhibited significantly lower WGR and SGR, and those under high-density stress also showed significant reductions in WGR, SGR, ADR, FER than the Control_2 group (*p* < 0.05).

**Table 1:** Growth performance of sea cucumber under normal and different stressful conditions.

Group		SR (%)	Final weight (g)	WGR (%)	SGR (%·d^−1^)	ADR (%)	FER (%)
Rearing 30 days	Control_1	100 ± 0	28.77 ± 0.21	59.96 ± 1.74	1.57 ± 0.04	36.46 ± 1.25	28.60 ± 1.71
	High temperature	100 ± 0	22.53 ± 0.18^***^	23.68 ± 1.56^***^	0.71 ± 0.04^***^	10.28 ± 2.20^***^	11.19 ± 0.91^***^
	Low salinity	100 ± 0	26.74 ± 1.12^*^	46.51 ± 5.78^*^	1.27 ± 0.13^*^	32.46 ± 0.70^**^	18.11 ± 1.83^**^
	NH_4_^+^-N	100 ± 0	27.36 ± 0.95	51.65 ± 4.70^*^	1.39 ± 0.10^*^	25.91 ± 1.84^**^	21.07 ± 1.81^**^
	NO_2_^−^-N	100 ± 0	25.12 ± 0.70^**^	40.91 ± 4.18^**^	1.14 ± 0.10^**^	20.28 ± 2.20^***^	20.84 ± 1.88^**^
Rearing 60 days	Control_2	100 ± 0	41.08 ± 1.18	128.19 ± 6.31	1.37 ± 0.05	37.81 ± 1.26	30.36 ± 3.00
	Starvation	100 ± 0	14.11 ± 0.79^***^	−22.02 ± 5.10^***^	−0.42 ± 0.11^***^	—	—
	High density	100 ± 0	34.40 ± 1.55^**^	92.28 ± 8.95^**^	1.09 ± 0.08^**^	27.21 ± 2.27^**^	23.43 ± 1.67^*^

Data represent mean ± S.E. Student’s *t*-test was employed to compare the differences between the control group and various environmental stress groups, and asterisks stand for significant differences (Student’s *t*-test: * represents *p* < 0.05, ** represents *p* < 0.01, *** represents *p* < 0.001).

### 3.2 Antioxidant and immunity capacity

**Figure 1:**
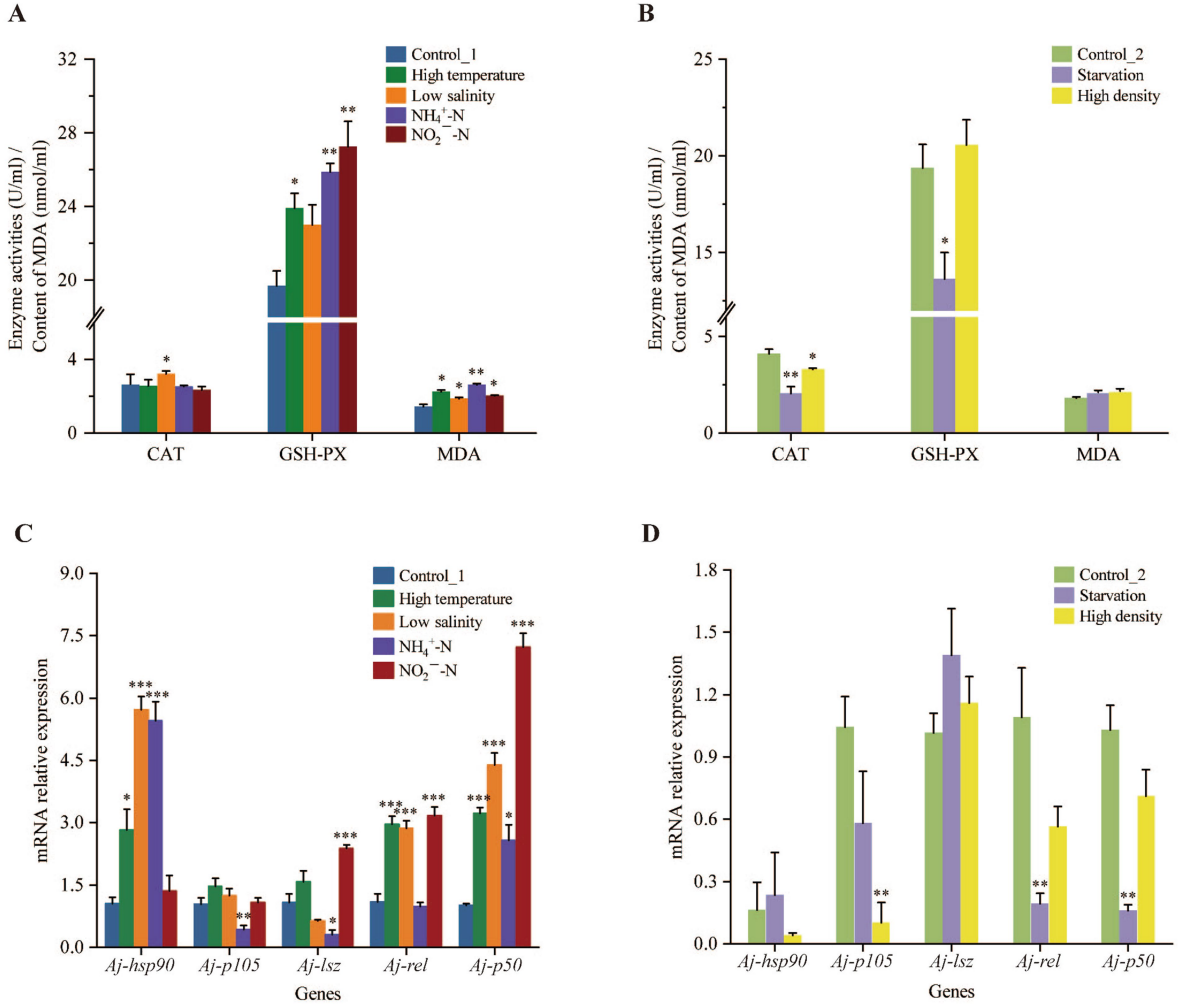
The antioxidant capacity **(A,B)** in coelomic fluid and relative expression levels of immune-related genes **(C,D)** in the mid-intestine of sea cucumber under different stressful conditions. The indicators related to antioxidant capacity were catalase (CAT) and glutathione peroxidase (GSH-PX) activities, and malondialdehyde (MDA) concentration. Data represent mean ± S.E. (*n* = 5). Asterisks stand for significant differences (determined by a two-tailed Student’s *t*-test: * represents *p* < 0.05, ** represents *p* < 0.01, *** represents *p* < 0.001).

The assessment of oxidative stress response based on the CS detection revealed that, after a 30-day pressuring period, the MDA content and GSH-PX activity exhibited an obvious increase in all four environmental stress treatments (High temperature group, Low salinity group, NH_4_^+^-N group, and NO_2_^−^-N group) compared to their control groups. Similarly, CAT activity in the Low salinity group was also significantly higher than in the Control_1 group (*p* < 0.05) (Figure 1A). However, compared to these four stress groups with a 30-day culture period, the High density group and Starvation group exhibited different performances. CAT activity significantly decreased in both two groups after a 60-day pressuring period (*p* < 0.05), and GSH-PX activity also significantly declined under the long-term starvation stress, compared to their control groups (*p* < 0.05). While the MDA content under both stressful conditions showed a similar level of the Control_2 group (*p* > 0.05) (Figure 1B).

Furthermore, sea cucumbers exposed to thermal, hypoosmotic, ammonium, and nitrite stress showed significant upregulation of two or more immune-related genes in the mid-intestine compared to the Control_1 group (*p* < 0.05) (Figure 1C). Specifically, in the High temperature group and Low salinity group, *Aj-hsp90*, *Aj-rel*, and *Aj-p50* were significantly upregulated (*p* < 0.05). There also was an observation in the NO_2_^−^-N group that *Aj-lsz*, *Aj-rel*, and *Aj-p50* expression showed significantly up-regulated than the Control_1 group (*p* < 0.05). Furthermore, in the NH_4_^+^-N group, *Aj-hsp90* and *Aj-p50* expressions were significantly up-regulated (*p* < 0.05), while *Aj-rel* and *Aj-p105* expressions were significantly down-regulated (*p* < 0.05). However, in contrast to the performance of the aforementioned four treatment groups, the down-regulated mRNA expression patterns of partial immune genes were revealed in the 60-day stress groups (Figure 1D). Specifically, *Aj-rel* and *Aj-p50* exhibited significant decreases in relative expression levels in the Starvation group (*p* < 0.05), while *Aj-p105* expression showed a significant down-regulated in the High density group (*p* < 0.05).

### 3.3 Changes in intestinal microbiota in sea cucumber under various environmental stress

#### 3.3.1 Intestinal microbiota diversity of sea cucumber

After quality control and paired-end merging, about 1,624,755 valid sequences, averaging 421 bp in length, were derived from the raw sequences of 50 intestinal microbiota sequencing samples GenBank accession PRJNA1156246. Additionally, the rarefaction curve analysis demonstrated the sequencing depth of these samples was adequate, allowing for further analysis (Supplementary Figure S1).

**Figure 2:**
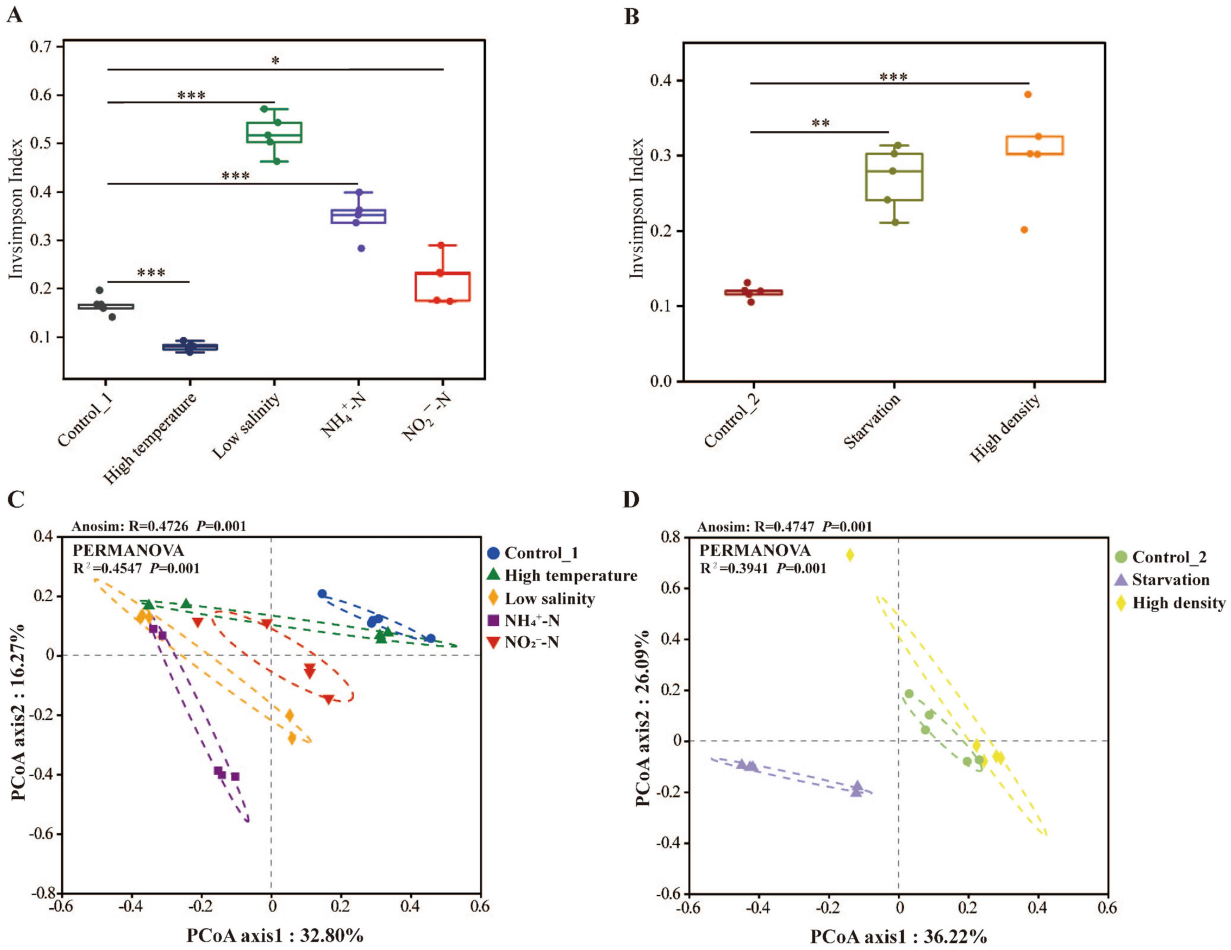
Effects of different environmental stress on the diversity of sea cucumber intestinal microbiota. **(A,B)** Inverse Simpson index (Student’s *t*-test: * represents *p* < 0.05, ** represents *p* < 0.01, *** represents *p* < 0.001); **(C,D)** Principal coordinate analysis (Adonis and Anosim tests, based on Bray-Curtis distance).

The alterations in the alpha diversity and structure of sea cucumber intestinal microbiota under various environmental stresses were assessed (Figure 2; Table 2). Specifically, the Invsimpson index of the Control_1 group was significantly lower than that of the Low salinity group, NH_4_^+^-N group, and NO_2_^−^-N group, but higher than that of the High temperature group (*p* < 0.05) (Figure 2A). Moreover, the Control_2 group exhibited a significantly lower inverse Simpson index compared to the Starvation and High density groups (*p* < 0.05) (Figure 2B). PCoA plot revealed the separation between different environmental stress groups and control groups (Figures 2C,D), ANOSIM and Adonis tests further revealed the differential intestinal microbiota structure in the five environmental stress groups compared to their respective control groups (*p* < 0.05), except for the High density group.

**Table 2:** One-way analysis of similarities (ANOSIM) and permutational MANOVA (PERMANOVA, Adonis) of intestinal microbiota structure from different environmental stress groups and control groups based on Bray-Curtis distance.

Group	ANOSIM		Adonis	
	*r*	*p*	*r* ^2^	*p*
High temperature vs. Control_1	0.492	0.011	0.343	0.021
Low salinity vs. Control_1	0.748	0.011	0.453	0.020
NH_4_^+^-N vs. Control_1	0.932	0.011	0.556	0.021
NO_2_^−^-N vs. Control_1	0.580	0.011	0.377	0.020
Starvation vs. Control_2	0.756	0.019	0.431	0.013
High density vs. Control_2	0.036	0.296	0.126	0.262

#### 3.3.2 Intestinal microbiota composition of sea cucumber

**Figure 3:**
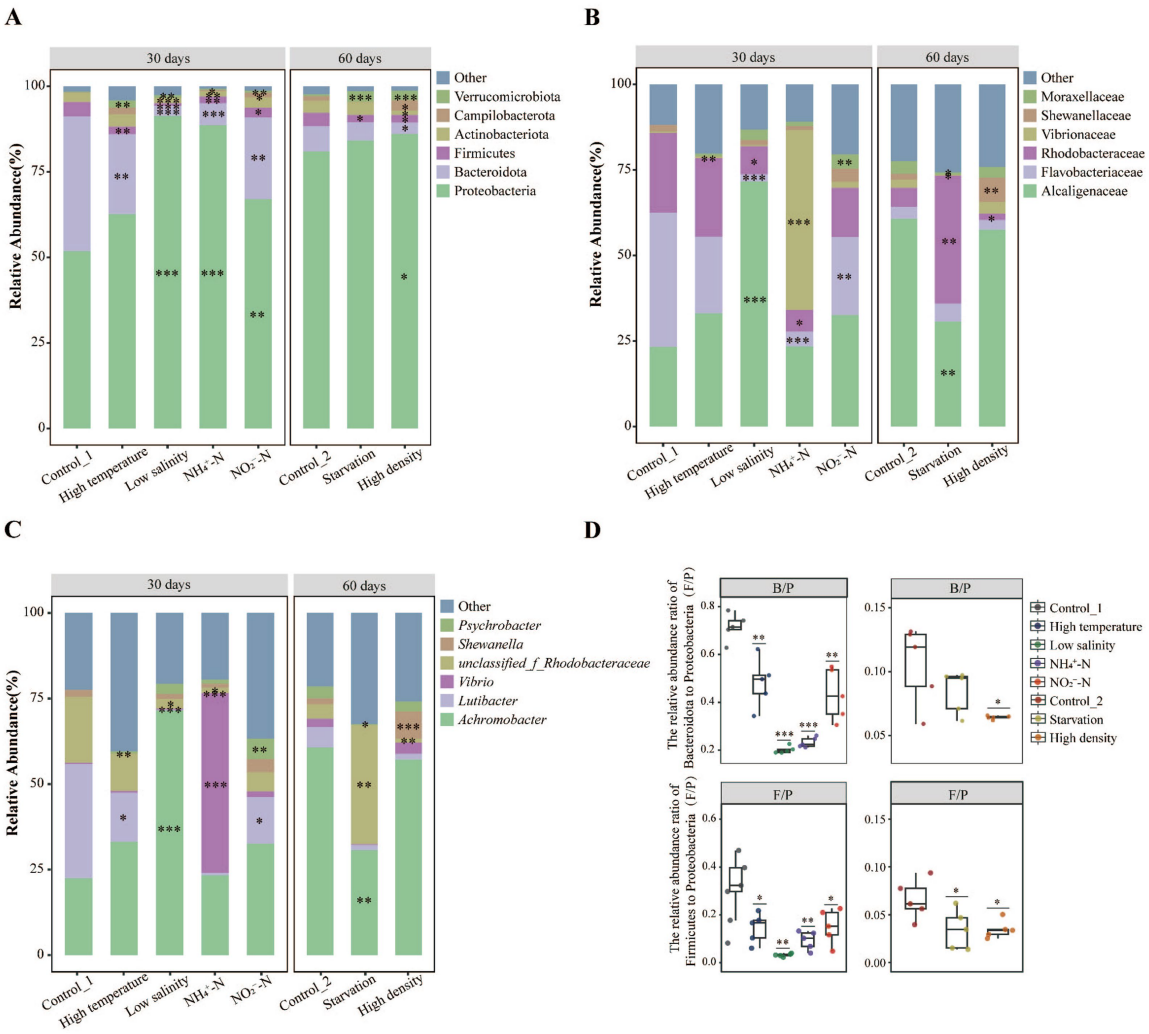
Effect of different environmental stressors on the intestinal microbiota composition of sea cucumber at **(A)** phylum, **(B)** family, **(C)** genus levels (top 6), and **(D)** differences in the relative abundance ratios of Bacteroidota/ Proteobacteria (B/P) and Firmicutes/ Proteobacteria (F/P) between environmental stress groups and control groups (Student’s *t*-test: * represents *p* < 0.05, ** represents *p* < 0.01, *** represents *p* < 0.001).

The analysis of intestinal microbiota composition revealed significant changes in the dominant (top six) phyla (Figure 3A), families (Figure 3B), and genera (Figure 3C) among the six environmental stress groups compared to control groups. Specifically, compared to their respective control groups, the relative abundance of Firmicutes (0.7–2.8%) and Bacteroidota (3.0–23.9%) significantly decreased in all six environmental stress groups and stress groups excluding the Starvation group (*p* < 0.05). While the relative abundance of Verrucomicrobiota (0.7–3.1%) significantly increased in stress groups excluding NH_4_^+^-N group (*p* < 0.05). Furthermore, compared to their respective control groups, the relative abundance ratios of Firmicutes to Proteobacteria (F/P) and Bacteroidota to Proteobacteria (B/P) significantly decreased in all aquaculture environmental stress groups and stress groups excluding the Starvation group, respectively (*p* < 0.05) (Figure 3D).

At the family level, compared to their respective control groups, significant decreases in the relative abundance of Flavobacteriaceae and Rhodobacteraceae were observed in the Low salinity and NH_4_^+^-N groups (*p* < 0.05). However, significant increases in the relative abundance of Alcaligenaceae, Vibrionaceae, Moraxellaceae, Rhodobacteraceae, and Shewanellaceae were observed in the Low salinity, NH_4_^+^-N, NO_2_^−^-N, Starvation, and High density groups, respectively (*p* < 0.05). Similar changes in intestinal microbiota composition were also observed at the genus level, the significant increases in the relative abundance of *Achromobacter*, *Vibrio*, *Psychrobacter*, *unclassified_f_Rhodobacteraceae*, and *Shewanella* were observed in the Low salinity, NH_4_^+^-N, NO_2_^−^-N, Starvation, and High density groups, respectively (*p* < 0.05). Moreover, a significant decrease in the relative abundance of *Lutibacter* in the 30-day stress groups (*p* < 0.05) was also observed.

#### 3.3.3 Key microbial characteristics for distinguishing whether sea cucumbers are under stress

**Figure 4:**
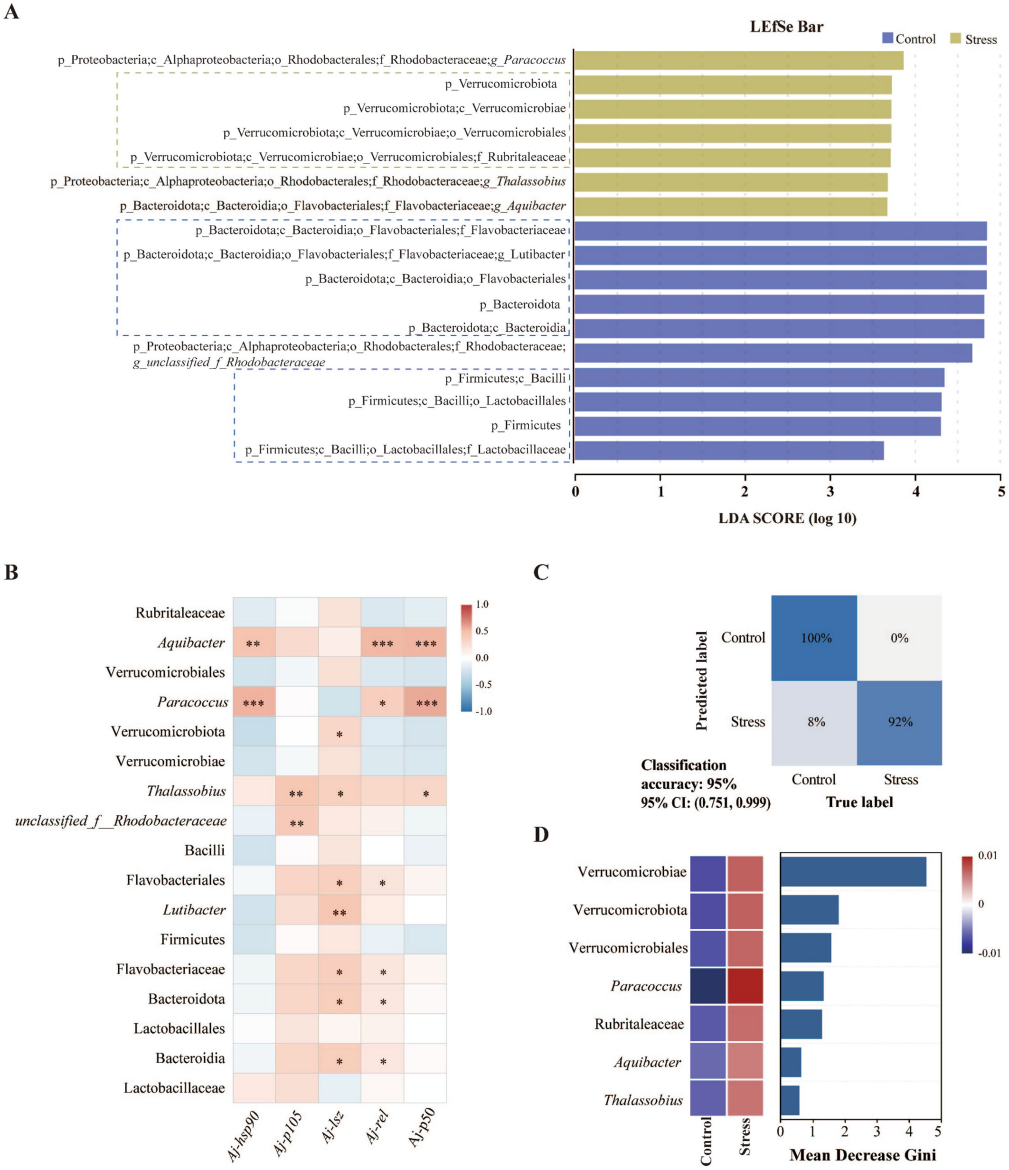
Prediction of stress and non-stress states in sea cucumber using microbiota. **(A)** LEfSe analyses to identify the most differential taxa by comparing stressed groups with non-stressed controls (LDA score > 3.5, *p* < 0.05); **(B)** A heat map of Spearman correlation coefficients was composed for the abundance of the most differential taxa and relative expression of immune-related genes in the intestine of sea cucumber (the correlation *p*-values were adjusted by Benjamini & Hochberg method, * represents *p* < 0.05, ** represents *p* < 0.01, *** represents *p* < 0.001); **(C)** Accuracy of the random forest model in predicting stress and non-stress states in sea cucumber; **(D)** Abundance distribution (centralized) and importance of key taxa identified by the random forest analysis in distinguishing stressed from non-stressed states in sea cucumber.

The indicator species analysis results showed that the number of significant indicator species unique to each experimental group is much higher than the number shared among groups, and the proportion of significant indicator species shared among the stressed groups (12.40%) was greater than that shared between the control group and the stressed groups (8.54%) (Supplementary Figure S2). Therefore, LEfSe analysis was used to identify the most differential taxa between all non-stress samples (Control group) and all stress samples (Stress group) at different cultivation times, to determine the microbial characteristics that best explained differences between groups (Figure 4A). A total of 17 different taxa ranging from phylum to genus level were obtained, of which, significant enrichment was observed in seven taxa in the Stress group and ten taxa in the Control group. Among these, the Rubritaleaceae (belonging to the Verrucomicrobiota, Verrucomicrobiae, and Verrucomicrobiales) was significantly enriched in the Stress group at the phylum, class, order, and family levels (*p* < 0.05). While the *Lutibacter*, which belongs to the Bacteroidetes, Bacteroidia, Flavobacteriales, and Flavobacteriaceae was significantly enriched in the Control group across all taxonomic levels (*p* < 0.05). Additionally, the Lactobacillaceae, which belongs to the Firmicutes, Bacilli, and Lactobacillales was also significantly enriched in the Control group across all taxonomic levels (*p* < 0.05).

The Spearman correlation test was further conducted to examine the relationship between the relative abundance of 17 differential taxa as described above and the relative expression levels of intestinal immunity-related genes (Figure 4B). The results indicated that 10 different species were significantly positively correlated with at least one immune gene’s relative expression level. Notably, the relative abundance of *Aquibacter* and *Paracoccus*, which were significantly enriched in the Stress group, showed significant positive correlations with the expression levels of *Aj-hsp90*, *Aj-rel*, and *Aj-p50*. The relative abundance of *Thalassobius* which was also significantly enriched in the Stress group was significantly positively correlated with the expression levels of *Aj-p105*, *Aj-lsz*, and *Aj-p50*.

Furthermore, based on the LEfSe analysis results, seven differential taxa significantly enriched in the Stress group was selected for random forest analysis (these taxa were significantly correlated with sea cucumber health status as verified by random forest, Supplementary Figure S3). The confusion matrix test results indicated that the intestinal microbiota of sea cucumbers can be used to predict their health status (stressed/non-stressed) with an accuracy of 95% (Figure 4C). Additionally, the top three microbial features that contributed significantly to distinguishing different health statuses of sea cucumber were identified as Verrucomicrobiae, Verrucomicrobiota, and Verrucomicrobiales (Figure 4D).

#### 3.3.4 Ecological network of intestinal microbiota

**Figure 5:**
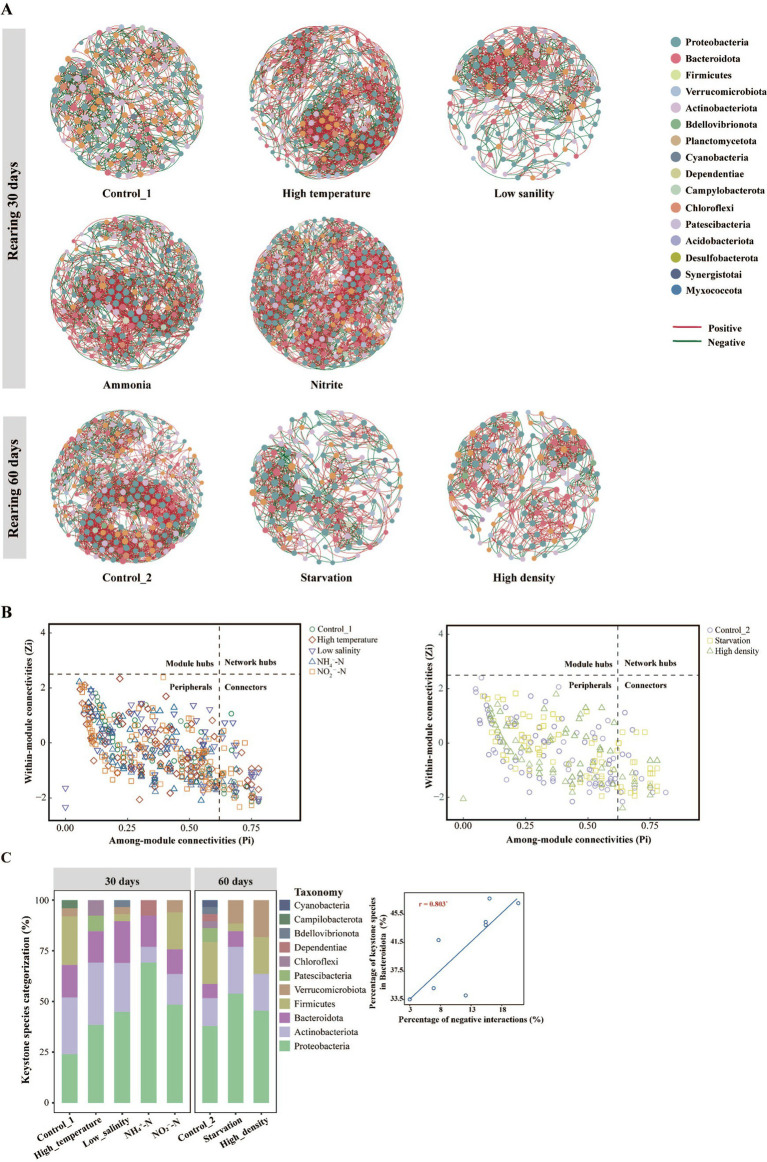
The co-occurrence networks analysis and the distribution of topological roles of intestinal microbiota in sea cucumber. **(A)** Co-occurrence networks of bacterial OTUs, and node colors represent phylum level classification, the red and green edges indicating positive and negative interactions between species; **(B)** Distribution of topological roles, the topological role of each node was determined according to the scatter plot of within-module connectivity (Zi) and among-module connectivity (Pi), peripherals: Pi <0.62 and Zi < 2.5, connectors: Pi >0.62, modules hubs: Zi > 2.5, network hubs: Pi >0.62 and Zi > 2.5; **(C)** Categorization of network connectors at phylum level and Pearson correlation between the proportion of network connectors belonging to Bacteroidota and the proportion of negative interactions (the correlation *p*-values were adjusted by Benjamini & Hochberg method, * represents *p* < 0.05).

As shown in Figure 5A, in reared 30-day groups, the co-occurrence networks of Control_1, High temperature, Low salinity, NH_4_^+^-N, and NO_2_^−^-N groups consist of 184, 213, 135, 222, 298 nodes, and 1,067, 1767, 761, 1847, 2,874 edges, respectively. In reared 60-day groups, the co-occurrence networks of Control_2, Starvation, and High density groups consist of 247, 143, 149 nodes, and 2,441, 805, 826 edges, respectively. Notably, the Control_1 group had a higher proportion of network nodes that were classified as Firmicutes (29.89%) and a higher proportion of negative interactions (47.7%) than those of the four stress groups (7.66–12.21% classified as Firmicutes, 33.99–47.04% of negative interactions) (Table 3). Additionally, Control_2 also had a greater proportion of network nodes that classified as Firmicutes (17%) compared to the stress groups (9.79–16.11%), and the proportion of negative interactions (35.11%) was higher than the High density group (33.54%) but lower than Starvation group (41.86%) (Table 4). Topological roles analysis (Figure 5B) revealed that most OTUs in all group’s networks were classified as Peripherals, followed by Connectors that belong to key nodes (keystone taxa), and no other key nodes (like Module hubs and Network hubs) were identified. Specifically, in reared 30-day groups, there were 26, 14, 30, 14, and 34 Connectors in the networks of Control_1, High temperature, Low salinity, NH_4_^+^-N, and NO_2_^−^-N groups, and there was a higher proportion of Connectors that classified as Bacteroidota (12.12–20.69%) (Figure 5C); In reared 60-day groups, there were 30, 27, and 12 Connectors in the networks of the Control_2, Starvation and High density groups, and about 3.01–7.69% of Connectors were classified as Bacteroidota. The Pearson correlation analysis further revealed a significant positive correlation (*p* < 0.05) between the proportion of network connectors belonging to Bacteroidota and the proportion of negative interactions (Figure 5C).

**Table 3:** Topological properties of intestinal microbiota in sea cucumber under different stressful conditions (rearing for 30 days).

	Control_1	High temperature	Low salinity	NH_4_^+^-N	NO_2_^−^-N
Nodes	184	213	135	222	298
Edges (negative/positive)	1,067 (509/558)	1767 (777/990)	761 (358/403)	1847 (820/1027)	2,874 (977/1897)
Clustering coefficient	0.571	0.599	0.601	0.583	0.539
Network density	0.063	0.078	0.084	0.075	0.065
Network diameter	8	8	13	7	8
Average degree	11.598	16.592	11.274	16.640	19.289
Modularity	0.573	0.600	0.540	0.583	0.580
Average path length	3.800	3.747	4.398	3.608	3.568

**Table 4:** Topological properties of intestinal microbiota in sea cucumber under different stressful conditions (rearing for 60 days).

	Control_2	Starvation	High density
Nodes	247	143	149
Edges (negative/positive)	2,441 (857/1584)	805 (337/468)	826 (277/549)
Cluster coefficient	0.599	0.610	0.621
Network density	0.080	0.079	0.075
Network diameter	7	9	11
Average degree	19.765	11.259	11.087
Modularity	0.554	0.596	0.651
Average path length	3.618	3.976	4.358

Furthermore, when investigating the topological properties of each network (Tables 3, 4), the Control_1 group displayed noticeably lower network clustering coefficients, densities, average degrees, and modularity in comparison to most stress groups (three or more groups). Similarly, in the 60-day group, the Control_2 group had lower topological properties (clustering coefficient, diameter, modularity, average path length) as compared to the two stress groups.

#### 3.3.5 Intestinal microbiota function of sea cucumber

**Figure 6:**
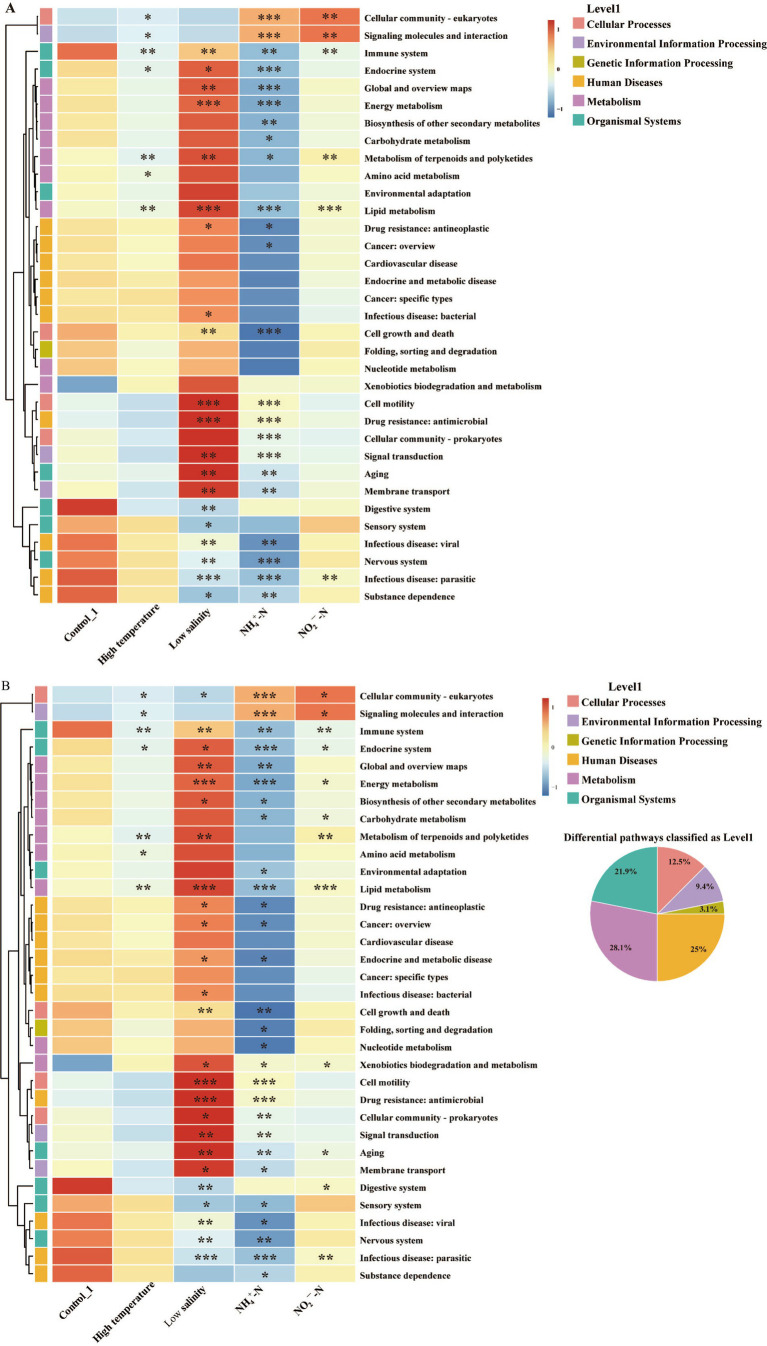
Comparison of intestinal microbiota function in sea cucumber between environmental stress groups and control groups based on KEGG level 2 pathways (top34) (Student’s *t*-test: * represents *p* < 0.05, ** represents *p* < 0.01, *** represents *p* < 0.001). **(A)** Groups rearing for 30 days, **(B)** groups rearing for 60 days.

The potential functions of the intestinal microbiota in stressed sea cucumber were predicted by PICRUSt2 (Figure 6). As shown in Figure 6A, 7, 20, 23, and 6 significantly different functional pathways observed in the High temperature, Low salinity, NH_4_^+^-N, and NO_2_^−^-N groups, respectively. However, in the Starvation group, only 6 differential functional pathways were annotated compared to the Control_2 group, while no differential functional pathways were observed between the High density group and Control_2 group (Figure 6B). Specifically, compared with the corresponding control group in different rearing periods, the immune system pathway showed a significant decrease in all stress groups except the High density group (*p* < 0.05). Signaling molecules and interaction pathways, as well as metabolic pathways including lipid metabolism and metabolism of terpenoids and polyketides, also exhibited significant differences in at least four stress groups (*p* < 0.05). Additionally, compared to the Control_1 group, multiple metabolism-related pathways, including energy metabolism, lipid metabolism, and the metabolism of terpenoids and polyketides, were significantly enriched in the Low salinity group (*p* < 0.05) but significantly decreased (*p* < 0.05) in the NH_4_^+^-N group.

## 4 Discussion

Induced by global climate change and unsustainable aquaculture practices, common aquacultural environmental stresses (such as fluctuations in water temperature and salinity, accumulation of toxic compounds like ammonium and nitrite, and limitations in farming space and food resources) can disrupt the equilibrium of intestinal microbiota in aquatic species, leading to an ecological imbalance that increases the host’s susceptibility to pathogenic bacteria (Petersen and Round, 2014). Consequently, researchers have extensively compared gut microbiome-host interactions under normal and various stressful conditions to identify common microbial characteristics linked to intestinal dysbiosis in aquaculture animals (Infante-Villamil et al., 2021; Xavier et al., 2023). However, there remains a paucity of such comprehensive studies on the economically significant species like the sea cucumber. Therefore, this study investigated the effects of thermal, hypoosmotic, ammonium, nitrite, crowding, and starvation stress on the growth, antioxidant, and immune defenses of the sea cucumber, and further attempts to summarize the common and differential microbial characteristics of intestinal microbial dysbiosis in sea cucumber under different stresses.

Physiological variations such as growth and digestive performance, in aquatic species can reflect their adaptive capacity to specific environments (Duan et al., 2017; Limbu et al., 2018; Xie et al., 2023). In this study, a significant negative growth in the body weight of sea cucumber was observed after a 60-day fasting period. Moreover, when under diverse stressful conditions that included a temperature of 23°C, salinity of 22, 0.5 mg/L NH_4_^+^-N, 0.25 mg/L NO_2_^−^-N, or a stocking density of 6 kg/m^3^ after 30 or 60 culturing days, there was a notable impairment in the growth performance (as indicated by the WGR and SGR) and digestive capability (as represented by the ADR and FER) of sea cucumber across all experimental groups. These findings suggest the significant inhibition in the physiological process of sea cucumbers under these six stressful conditions.

As a vital component of the sea cucumber’s nonspecific immune system, coelomocytes produce and release large amounts of reactive oxygen species (ROS) and bactericidal humoral factors to enhance immune defense against pathogens and environmental stimuli (Wu et al., 2020). However, prolonged exposure to stress can trigger an oxidative stress response in aquatic species, disrupting the balance between ROS production and removal by the antioxidant system (comprised of various cytokines and antioxidant enzymes), thus resulting in oxidative damage (Li et al., 2023; Zhao Y. et al., 2023). Hence, within the 30-day exposure groups, the substantial increase in the activity of the antioxidant enzyme CAT under hypoosmotic stress, and the augmentation of antioxidant enzyme GSH-PX activity under thermal, ammonium, and nitrite stress, indicating excessive ROS production in coelomocytes under these conditions (Huo et al., 2019a). The marked increase in MDA concentration further reflects lipid peroxidation due to ROS accumulation, leading to peroxidative damage to coelomocytes (Lushchak, 2011). In contrast, persistent fasting stress significantly decreased CAT and GSH-PX activities, likely due to the adverse effects of sustained fasting on the physiological condition of sea cucumbers, thereby weakening their oxidative defense capabilities (Llesuy et al., 2001). Additionally, the minor changes in antioxidant indices observed in sea cucumbers under high-density stress may be attributed to a relatively weaker stress effect that are insufficient to elicit a strong oxidative stress response (Wang et al., 2019).

Furthermore, the imbalance of intracellular redox homeostasis induced by stress can lead to the disruption of the intestinal barrier function in sea cucumbers, and further triggering inflammatory responses and other immune defense processes mediated by the nuclear factor-κB (NF-κB) signaling pathway (Wang et al., 2013; Zhao et al., 2019). Consistently, within the 30-day exposure groups, an overall upregulation trend in the expression of *Aj-rel* and *Aj-p50*, genes involved in the NF-κB signaling pathway, was observed in the mid-intestine of sea cucumber. In contrast, *Aj-p105* expression showed no significant difference or a decrease compared to the control group, suggesting that stress stimulation may have induced the degradation of Aj-p105 protein and the nuclear translocation of Aj-rel and Aj-p50 proteins (Wang et al., 2013). Additionally, the heat shock protein coding gene (*Aj-hsp90*) exhibited an upregulation trend in the aforementioned stress groups to protect proteins from oxidative damage and maintain normal cellular physiological functions. These results indicate that the NF-κB signaling cascade and HSPs-related genes are critical for inflammatory and immune responses under different stressful conditions (Huo et al., 2019b). The significant changes in NF-κB pathway gene expression under starvation and crowding stress further highlight its pivotal role in the sea cucumber’s adaptive response to diverse stress. Additionally, due to the flexible nature of lysozyme-induced immune defense in sea cucumbers under stress, variability was observed in the expression of the lysozyme-encoding gene (*Aj-lsz*) among different stress groups (Huo et al., 2019b).

Previous research has demonstrated that the crosstalk between the innate immune system of aquatic animals and their intestinal microbiota is beneficial for enhancing the host’s immune responses and maintaining the stability of the intestinal microbial ecosystem (Cao et al., 2021). The significant correlation between the expression levels of lectin-encoding genes in the respiratory tree of sea cucumber and the relative abundance of *Flavirhabdus* was revealed by Song et al. (2019). Similarly, research by Yang et al. (2015) demonstrated that the enrichment of Rhodobacteraceae enhanced the expression of the NF-κB signaling pathway, thereby strengthening the immune defense of the sea cucumber. Consistently, a significant correlation between the relative abundance of most differential species (identified by LDA analysis) between stressed and healthy sea cucumbers and the expression levels of intestinal immune-related genes was revealed in this study. Moreover, the function of intestinal microbiota involved in host immunity in five stress groups (excluding the High density group) was all weakened, which further highlights the close interrelationship between intestinal microbiota dysbiosis under environmental stress and the host’s immune defense functions. Therefore, it is critical to identify the characteristics of microbial imbalances in sea cucumbers under different stress and determine if these responses follow a consistent pattern, allowing for early intervention before the host reaches an irreversible disease state.

In the current study, the disruption of intestinal physiological homeostasis in sea cucumbers under various environmental stress-induced intestinal microbiota dysbiosis and altered intestinal microbial composition and structure (Duan et al., 2021). Specifically, these pressures result in disturbed redox homeostasis of intestinal environments, which could potentially lead to the accumulation of ROS and/or reactive nitrogen species (RNS), and might further lead to a surge proliferation of facultative anaerobic Proteobacteria and a decreasing abundance of obligate anaerobes, including specific taxa within Bacteroidota and Firmicutes (Winter and Bäumler, 2014). Therefore, there was a significant decline in the Bacteroidota: Proteobacteria (B/P) and Firmicutes: Proteobacteria (F/P) abundance ratios in each environmental stress group. Previous studies have shown that certain taxa within Bacteroidota (Karlsson et al., 2011) and Firmicutes (Sun et al., 2023) play a positive role in intestinal homeostasis regulation by participating in the fermentation of complex carbohydrates to modulate intestinal epithelial barrier function and cytokine production. Therefore, a decrease in B/P and F/P ratios often indicates alterations in intestinal microbiota homeostasis and may even indicate the occurrence of intestinal inflammatory responses (Xavier et al., 2023). In this study, it was also found that the healthy sea cucumbers exhibited a greater number of intestinal microbiota network nodes classified as Firmicutes. Additionally, a significant positive correlation was identified between the proportion of core network nodes classified as Bacteroidota and the proportion of negative interspecies interactions, which serve as indicators of microbiota network stability. These findings suggest that the reduced relative abundance of Bacteroidota and Firmicutes may diminish their positive roles in maintaining host intestinal health and suppressing the growth of harmful bacteria. This may induce the outbreak of opportunistic pathogens in the stressed sea cucumbers, such as Vibrionaceae (Xavier et al., 2023), Moraxellaceae (Welter et al., 2021), and Shewanellaceae (Zhang et al., 2016), and then alter the interspecies interactions within the intestinal microbiota network and affect network stability. These results underscore the critical role of Bacteroidota and Firmicutes in sustaining intestinal microbiota homeostasis (Ruzauskas et al., 2021; Xiong et al., 2017). Interestingly, alterations in the intestinal microenvironment also led to a significant enrichment of beneficial microorganisms, such as *Achromobacter* and Rhodobacteraceae, which are known to promote host growth and immune defense, they were particularly enriched under hypoosmotic and starvation stress, respectively (Wang et al., 2024; Zhang et al., 2020). In both environmental stress groups, relatively higher stability within the intestinal microbiota network was also obtained, which may reflect an adaptive response of intestinal microbiota in sea cucumber to these specific stress. A similar observation was also found in a previous study which revealed an increased stability of the intestinal microbiota in zebrafish exposed to silver nanoparticles (Chen et al., 2021).

The significant shifts in the abundance of these dominant taxa under the distinct stress influenced the survival niches of other taxa, ultimately leading to marked changes in microbiota diversity (Invsimpson index) within each stress group and an increase in microbiota network complexity. These findings of this study are consistent with those reported by Dai et al. (2020), which identified significant alterations in gut microbiota *α*-diversity associated with dysbiosis. These findings of this study are consistent with those reported by Dai et al. (2020), which identified significant alterations in gut microbiota α-diversity associated with dysbiosis. However, they contrast with findings from studies that consistently observed reductions in both community diversity and complexity under dysbiosis (Barko et al., 2018; Rosenfeld, 2017). These discrepancies may reflect variations in the intestinal microbiota’s responses to different types and intensities of stressors examined across studies. Moreover, it was revealed that the High density group, which might experienced relatively mild stress, did not exhibit the significant alterations in intestinal microbiota structure observed in the other stress groups, in the present study. This finding also underscores the importance of considering both the type and intensity of stress when using significant microbiota structural changes as indicators of ecological dysbiosis in aquatic animals (Xavier et al., 2023). PICRUSt analyses further revealed that organisms adopt distinct/ similar strategies to cope with different stress, different stress drives differential/ consistent functional responses of intestinal microbiota while all consistently exhibit a diminished role in host immunity. For example, under hypoosmotic stress, the intestinal microbiota adapted to environmental changes and maintained basic cellular functions by increasing energy production, adjusting membrane lipid composition, and secreting antioxidants, which collectively led to a significant upregulation in metabolic pathways related to energy metabolism, lipid metabolism, and the metabolism of terpenoids and polyketides (Xie et al., 2023). While, under the chemical stresses of ammonium or nitrite, the intestinal bacterial community of sea cucumbers showed increased involvement in pathways related to cellular communities and signaling interactions, which suggests that the intestinal microbiota enhances interactions within the microbial community and with host cells to cope with environmental stress (Duan et al., 2024). These findings align with the significant indicator species analysis, which indicated that the ammonium and nitrite stress groups shared a significant number of indicator species, accounting for 47.22 and 34.00% of the total identified in each group, respectively. Among these shared species unique to these two stress groups, 29.63% were classified as Flavobacteriaceae, known for promoting carbohydrate utilization, and 11.11% were classified as Sphingomonadaceae, which help regulate intestinal immune responses and maintain gut homeostasis by modulating natural killer T (NKT) cell functions (Oh et al., 2021; Zhang H. et al., 2019).

Under environmental stress, significant changes in the relative abundance of certain beneficial microorganisms or potential pathogens can serve as sensitive indicators for diagnosing the host’s health status. Therefore, the differential species between stressed and non-stressed sea cucumbers were further compared, aiming to identify diagnostic biomarkers that distinguish between stressed and normal intestinal microbiota. LEfSe analysis revealed that the maintenance of intestinal microbial homeostasis in healthy sea cucumbers largely depends on key taxa belonging to Bacteroidota and Firmicutes, such as *Lutibacter* and Lactobacillaceae, which secrete complex polysaccharide-degrading enzymes and further produce short-chain fatty acids (Barbato et al., 2022; Sartor and Wu, 2017), as well as unclassified_f_Rhodobacteraceae, which enhances the non-specific immune system of sea cucumbers (Yang et al., 2015). Interestingly, in stressed sea cucumbers, along with the enrichment of dysbiosis biomarkers like *Paracoccus* (Sheu et al., 2018) and potential pathogens such as *Aquibacter* (Kopprio et al., 2021) and *Thalassobius* (Tapia-Paniagua et al., 2018), it was also observed that Rubritaleaceae, which belongs to Verrucomicrobiota, Verrucomicrobiae, and Verrucomicrobiales, was significantly enriched at all taxonomic levels (Quan et al., 2019; Zhao Z. L. et al., 2023). In the previous studies, Verrucomicrobia species have also been identified as the significant microbial feature in the gut of grass carp (*Ctenopharyngodon idella*) and sea cucumbers under nanoplastics stress, and zebrafish exposed to combined polyethylene and 9-Nitroanthracene stress (Li et al., 2023; Zhang et al., 2021; Zhao Z. L. et al., 2023). This might be associated with the important role of Verrucomicrobia species in alleviating chronic low-grade inflammation and enhancing the intestinal barrier in dysbiotic host guts (Lindenberg et al., 2019; Zhao et al., 2024). Consistently, a random forest analysis based on these differential species combinations, validated by a confusion matrix with 95% accuracy, indicated that significant changes in the relative abundance of Verrucomicrobiae, Verrucomicrobiota, and Verrucomicrobiales are the most predictive features for identifying stressed sea cucumbers, further underscoring the potential of Verrucomicrobia species as diagnostic biomarkers for intestinal microbiota dysbiosis in aquatic animals.

## 5 Conclusion

This study revealed that under six different types, intensities, and durations of environmental stress stresses (including thermal, hypoosmotic, ammonium, nitrite, starvation, and crowding stress) that inhibited the growth and digestive capacity of sea cucumbers, the organisms exhibited varying degrees of oxidative stress response, which subsequently activated the NF-κB signaling pathway, and induced both common and unique features of intestinal microbiota dysbiosis. The critical roles of Bacteroidota and Firmicutes in maintaining intestinal microbiota homeostasis were demonstrated, while the significant enrichment of Verrucomicrobia species, particularly Verrucomicrobiae, as an important predictive indicator for distinguishing stressed individuals, was identified in this study. The findings indicated that significant alterations in intestinal microbiota diversity, notable decreases in the B/P and/or F/P ratios, and the enrichment of Verrucomicrobia species were common characteristics of intestinal microbiota dysbiosis in sea cucumber under various environmental stresses, these results would help establish efficient health monitoring strategies for sea cucumbers.

## Data Availability

The datasets presented in this study can be found in online repositories. The names of the repository/repositories and accession number(s) can be found below: https://www.ncbi.nlm.nih.gov/, PRJNA1156246.
