# Supplementary material for: Physiological and intestinal microbiota responses of sea cucumber Apostichopus japonicus to various stress and signatures of intestinal microbiota dysbiosis
Source: Front Microbiol. 2024 Dec 23;15:1528275. doi: 10.3389/fmicb.2024.1528275 (PMC11708840; doi:10.3389/fmicb.2024.1528275)
Supplement: Supplementary file 1 [file Supplementary_file_1.docx]

**Supplementary materials**

**Physiological responses of sea cucumber *Apostichopus japonicus* and their signatures of intestinal microbiota dysbiosis under different stressors in aquaculture**

Cui, Liang ^a^; Yumeng Xie ^a^^[[1]](#footnote-1)^*; Kai Luo ^a^; Mingyang Wang ^a^; Longzhen Liu ^a,b^; Changlin Li ^a^; Xiangli Tian ^a^^[[2]](#footnote-2)^*

*^a^The Key Laboratory of Mariculture (Ocean University of China), Ministry of Education,* *Qingdao 266000, P. R. China;*

*^b^The* *Yellow Sea Fisheries Research Institute, Chinese Academy of Fishery Sciences, Qingdao 266071, P. R. of China.*

**List of Table**

**Table S1** Primer sequences of internal reference and target genes for RT-qPCR.

**List of Figures**

**Fig. S1** Rarefaction curves of the intestinal bacterial community.

**Fig. S2** Bipartite networks illustrate the indicator species in the intestinal microbiota of sea cucumber across reared (A) 30-day and (B) 60-day groups. Nodes represent individual bacterial OTUs significantly associated (*P* < 0.05) with one or more treatments and node colors reflect their classification at the phylum level, edges indicate these associations.

**Fig. S3** Importance and singnifcance of differential microbes in distinguishing stressed from non-stressed states in sea cucumber. Asterisks stand for significant differences (* represents *P* < 0.05)

**Table S1** Primer sequences of internal reference and target genes for RT-qPCR.

| **Gene name** |  | **Primer sequence (5’-3’)** | **Source** |
| --- | --- | --- | --- |
| *β-actin* | Forward | TTATGCTCTTCCTCACGCTATCC | (Liu et al., 2022) |
|  | Reverse | TTGTGGTAAAGGTGTAGCCTCTCTC |  |
| *Aj-p50* | Forward | TCCTATCGGTCTGAATCTTCCAA |  |
|  | Reverse | TTTCTTCCCTTTCTGGCTATGTTC |  |
| *Aj-p105* | Forward | GCAACACACCCCTCCATCTT |  |
|  | Reverse | TCTTCTTCGCTAACGTCACACC |  |
| *Aj-rel* | Forward | TGAAGGTGGTATGCGTCTGG |  |
|  | Reverse | TTGGGCTGCTCGGTTATG |  |
| *Aj-lsz* | Forward | AGGGAGGTAGTCTGGATGGA | (Xu et al., 2024) |
|  | Reverse | GCGCAAAATCCTCACAGGTA |  |
| *Aj-hsp90* | Forward | GGAGGAGCGAACAAACCAAG |  |
|  | Reverse | GTCAAATGGCGCCCTCTTAG |  |


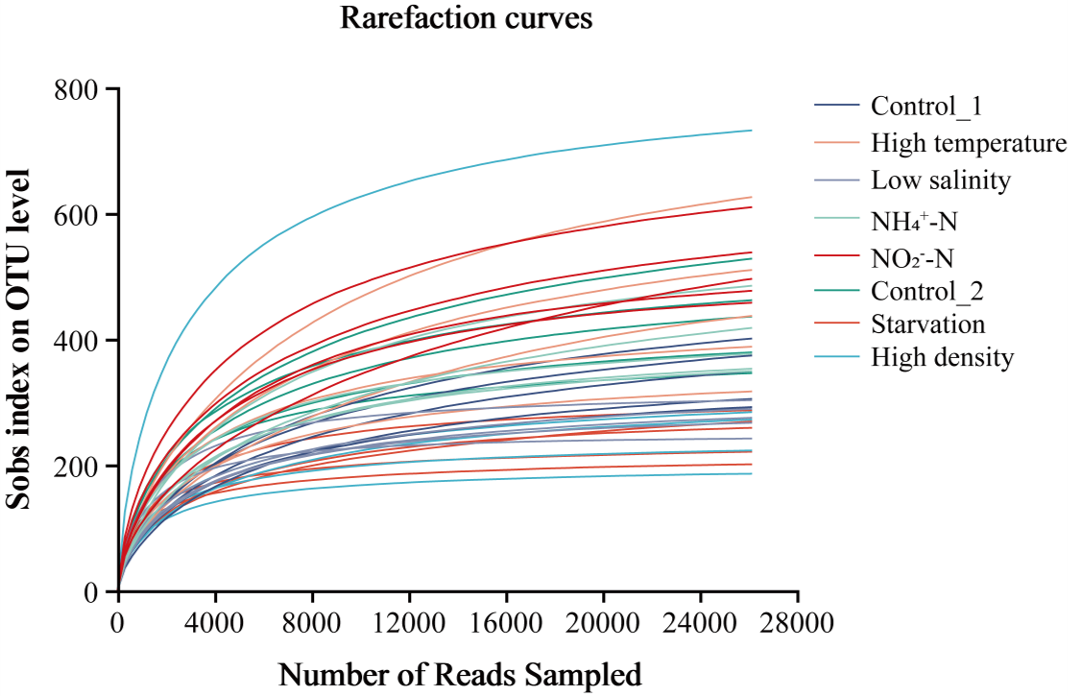


**Fig. S1** Rarefaction curves of the intestinal bacterial community.

**
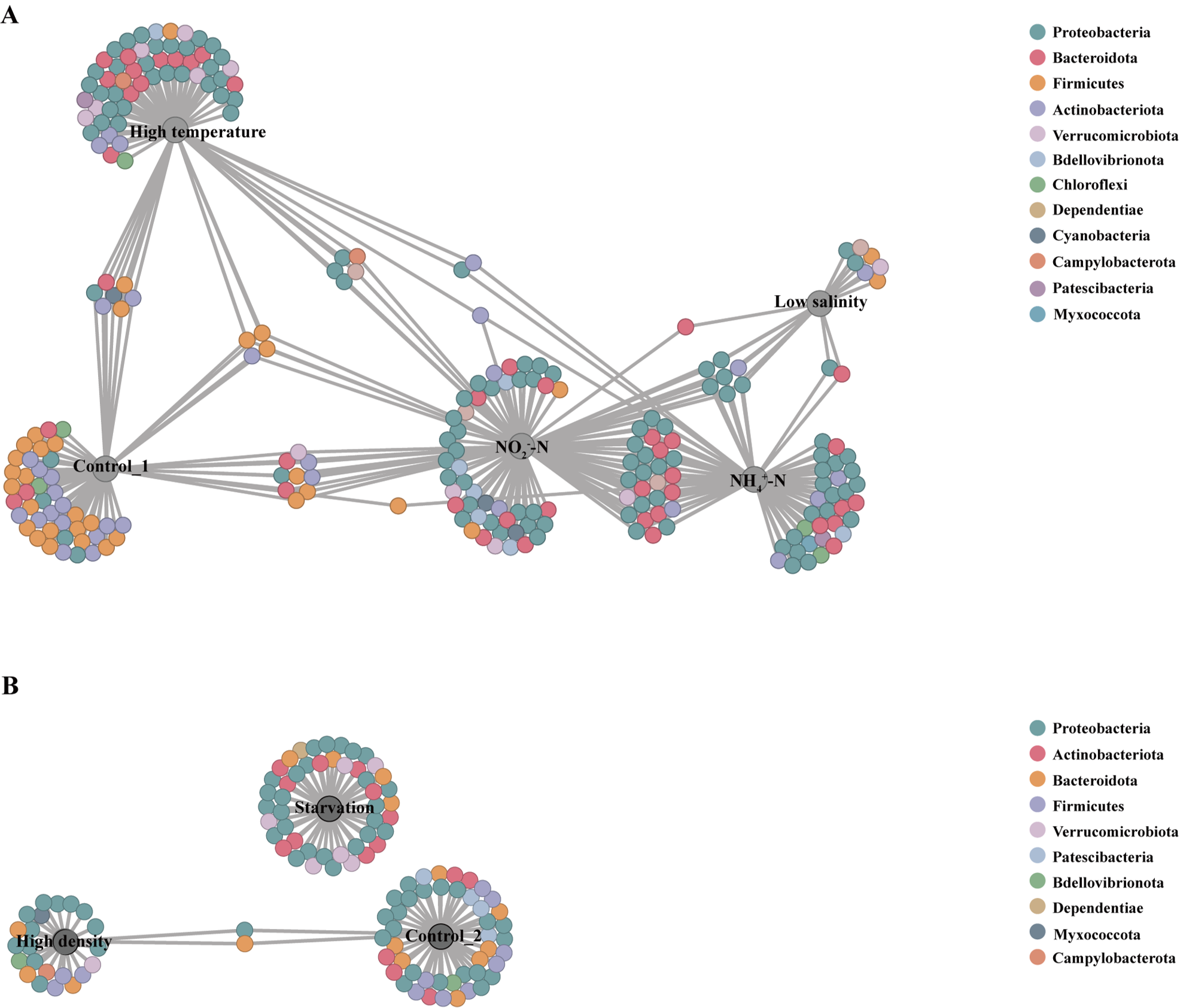
**

**Fig. S2** Bipartite networks illustrate the indicator species in the intestinal microbiota of sea cucumber across reared (A) 30-day and (B) 60-day groups. Nodes represent individual bacterial OTUs significantly associated (*P* < 0.05) with one or more treatments and node colors reflect their classification at the phylum level, edges indicate these associations.


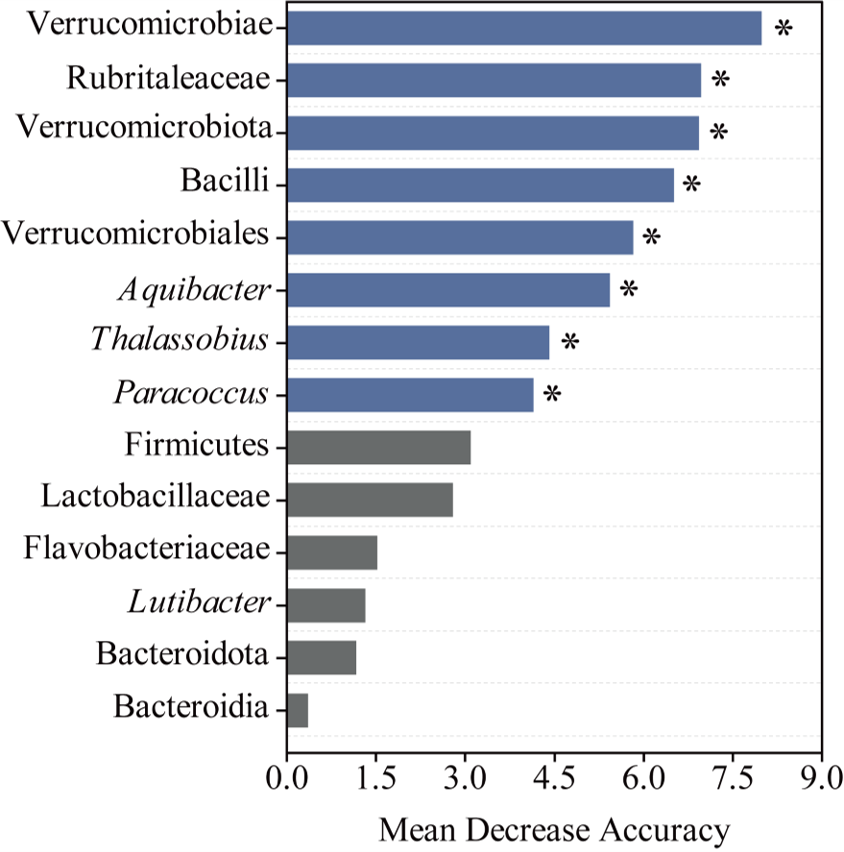


**Fig. S3** Importance and singnifcance of differential microbes in distinguishing stressed from non-stressed states in sea cucumber. Asterisks stand for significant differences (* represents *P* < 0.05).

1. ***** **Co-orresponding authors**

   Dr. Xiangli Tian

   Permanent address: 5# Yushan Road, Qingdao 266000, P. R. China.

   E-mail: xianglitian@ouc.edu.cn

   Dr. Yumeng Xie

   Permanent address: 106# Nanjing Road, Qingdao 266071, P. R. China.

   E-mail: yumeng21160511072@163.com [↑](#footnote-ref-1)
2. [↑](#footnote-ref-2)
